# Oral Function and Eating Habit Problems in People with Down Syndrome

**DOI:** 10.3390/ijerph19052616

**Published:** 2022-02-24

**Authors:** Sonia Cañizares-Prado, Jorge Molina-López, María Trinidad Moya, Elena Planells

**Affiliations:** 1 Department of Physiology, Faculty of Pharmacy, Institute of Nutrition and Food Technology “José Mataix”, University of Granada, 18071 Granada, Spain; sonia636@hotmail.es; 2 Granadown, Down Syndrome Association of Granada, 18014 Granada, Spain; mtmoru@yahoo.es; 3 Faculty of Education, Psychology and Sports Sciences, University of Huelva, 21007 Huelva, Spain

**Keywords:** down syndrome, intellectual disability, feeding problems, swallowing disorders, mastication, oral habits

## Abstract

Background: Down syndrome (DS) is a genetic disorder in which there is an increased risk of developing clinical comorbidities that require regular attention: health problems, alterations in maxillomandibular development, chewing and swallowing problems, as well as dietary habits that may influence diet and nutritional status. This study will analyze the frequency of occurrence of these factors with increasing age in this population. Methods: A descriptive cross-sectional study was conducted with 18 participants aged 30–45 years. The condition of orofacial structures, chewing and swallowing function and oral and eating habits were assessed to observe the frequency of occurrence of these problems with increasing age. Results: A high frequency of digestive problems was observed. There was also a presence of problems in the introduction of new tastes and consistencies. In addition, unilateral chewing was reported in 100% of the participants, severe anatomical dysfunction of the mandible/maxilla and high hypotonicity reflected in tongue movements. Conclusions: it is necessary to educate, through specific intervention protocols, the younger generations with DS, as well as their environment, as harmful habits are developed in childhood and consolidated throughout life.

## 1. Introduction

Down syndrome (DS), or trisomy 21, is a genetic disorder caused by the partial or complete presence of an extra copy of chromosome 21 [1]. The condition manifests with a variable degree of intellectual disability, developmental disturbances and characteristic physical features [2]. Etiologically, DS is associated with risk factors such as parental chromosomal abnormalities and/or advanced maternal age [3].

Once DS is diagnosed, it is assumed that there is an increased risk of developing multiple clinical comorbidities that require periodic medical attention, such as: congenital heart disease [4], thyroid dysfunction (one of the most common endocrine disorders associated with DS) [5], respiratory problems and obstructive sleep apnea [6], dysphagia (3.6%) [7] and gastroesophageal reflux disease (47%) [8]. In addition, there are specific anatomical characteristics of DS that complicate correct oral function at the maxillofacial level [9]. In this regard, the presence of reduced mandibular development stands out, which favors lingual protrusion, making lip occlusion difficult and often causes dental and swallowing problems [10]. Other common characteristics are labial and lingual hypotonia that hinder sucking, control and management of saliva and food bolus within the oral cavity [10]. The presence of an ogival palate and an altered and late dentition also stand out [10], leading to frequent dental agenesis [11] or the presence of supernumerary teeth that promote predominantly type III malocclusions [12]. All of these orofacial alterations cause swallowing difficulties [13], which can lead to serious health problems [14]. Such is the case that the airway can be compromised by the aforementioned hypotonia in adults with DS by presenting an insufficient crushing of food [15]. Regarding the oral phase of swallowing, patients with DS experience difficulties in keeping food in the oral cavity [16], which may be due to an impaired lip seal [17], intermaxillary discrepancy hindering the stabilization of the mandible and hyoid bone necessary for chewing and swallowing [18], as well as poor control and mobilization of the food bolus [10]. This, in turn, may be influenced by altered sensory perception, which may lead to an intolerance of certain food textures within the oral cavity [16]. The hypotonicity described above also exerts effects at the pharyngeal level, where weakened muscle strength increases the risk of aspiration before and after swallowing [19].

All of this has an impact on the eating habits of individuals with DS, with feeding difficulties estimated to prevail throughout life [20], if untreated. This results in the feeding behavior of adults with DS being characterized by a variable feeding rhythm [16], ranging from a very slow rhythm to an excessively fast rhythm that jeopardizes swallowing efficiency and safety, which favors digestive problems [21]. These problems are estimated to be present in 4.7% of adults with DS [7], with one of the predominant symptoms being constipation (4.1%) [7]. These dysfunctional eating habits are related to the obesity predominantly found in this population [22] and directly influence quality of life [8].

It is important to highlight the current scarcity of scientific literature focused on the study of the adult population with DS. Therefore, the need arises to focus specifically on the adult population and to avoid the generalization, commonly found, of findings obtained in the pediatric population. Based on the above, it was scientifically hypothesized that there would be a high frequency of problems related to oral function and eating habits, as well as poor myofunctional performance, influencing chewing and swallowing in adults with DS. Therefore, the present study aims to analyze the state of the maxillomandibular and oral structure, masticatory and swallowing performance and eating and dietary habits in a group of adults with DS from the GRANADOWN association (Granada, Spain).

## 2. Materials and Methods

### 2.1. Study Design

A cross-sectional descriptive study was carried out during the period from January to May 2019 at the “GRANADOWN” Down Syndrome Association in the city of Granada (Spain). The study complied with the ethical standards set out in the Declaration of Helsinki and with Spanish Organic Law 15/1999, of 13 December, on the Protection of Personal Data and was approved by the Ethics Committee of the University of Granada (862/CEIH/2019). Each participant received an explanation of the objective of the study, its duration and what participation would involve, and informed consent was obtained in all cases.

### 2.2. Participants

Non-probability purposive sampling was carried out involving 18 people with DS (8 women and 10 men), with a mean age of 34.5 ± 3.88 years, who are members of the association GRANADOWN. The participants had the genetic condition of DS, an age between 30–45 years, no prior myofunctional stimulation from early ages and were currently on an oral diet. In addition, subjects had to agree to participate in the study. Those individuals that failed to meet the above criteria were excluded from the study.

### 2.3. Procedure

The choice of study instruments was made by selecting those parameters that were important for the purpose of the study. Subsequently, assessment was carried out in 18 sessions, each with a duration of 50 min, in which the principal investigator evaluated each of the participants individually in the GRANADOWN speech therapy room during the breakfast schedule established by the association. To avoid measurement bias, the principal investigator was trained prior to measurement and supervised by the center’s speech therapists belonging to the research team.

### 2.4. Clinical History and Myofunctional Examination (MBGR)

This examination protocol was created and validated in 2012 by Marchesan et al. [23]. The protocol was created with the aim of collecting qualitative information regarding personal data, reason for consultation, family history, relevant data during pregnancy and birth, development and motor difficulties, health problems, respiratory problems, sleep, treatments, breastfeeding, difficulties in introducing drinking glass, flavors and consistencies, current diet, type of food predominantly eaten, places where it is eaten most of the time, chewing, swallowing, oral habits, postural habits and communication. The protocol was completed with the help of family members or people from the immediate environment of the participant. The variables included within each domain of the MBGR test were categorized according to the presence or absence of impairment: 0 indicated no impairment; 1 indicated the presence of sporadic or regular impairment.

### 2.5. Orofacial Myofunctional Assessment Protocol (OMES-E)

The OMES-E is a validated orofacial myofunctional assessment protocol with expanded scores (OMES-E) developed for assessment of the appearance, posture and mobility of the face, cheeks, jaw, tongue and lips, as well as of breathing, chewing and swallowing functions in terms of both performance and duration [24]. It makes use of a standardized food and a glass of water for swallowing. The overall scores range from 1–4 (4 = normal, 3 = mild dysfunction, 2 = moderate dysfunction and 1 = severe dysfunction). The data were recorded on video using a 12-megapixel camera placed one meter away from the participants and 0.85 m off the ground and supported by a Kentop tripod (Sachtler GmbH, Eching, Germany) measuring 185 × 42 × 36 mm. The measurements were taken in one of the GRANADOWN speech therapy rooms, with the participants sitting on a chair with a backrest. The recordings were made by the principal investigator and subsequently evaluated jointly by two members of the research team according to the study variables and the guidelines established by OMES-E. That is, they were scored taking into account that a higher score corresponded to better performance in the test. The time taken to eat the standardized food (cookie) was recorded with a stopwatch, starting from the moment the participant put the food in his or her mouth until the last swallow. Participants were only told when to put the food into the oral cavity to take the first bite, after which each participant performed chewing and swallowing in a natural way.

### 2.6. Heart Rate, Oxygen Saturation and Salivary pH

Heart rate (beats per minute (bpm)) and blood oxygen saturation (SaO_2_) were recorded before, during and after intake of the food and the glass of water. This was carried out using a MeasuPro pulse oximeter placed on the index finger of each participant. For salivary pH, each participant was given a test strip, which they had to place in direct contact with saliva. After 5 min, the color of the strip was checked against the equivalence chart to see the pH value, with 1 being very acidic and 14 very basic. The participants had not eaten any type of food and had not brushed their teeth during one hour before the measurement.

### 2.7. Data Analysis

The SPSS statistical package was used for data analysis. For this purpose, the median age of the participants was calculated to establish a cut-off point between two groups. On the one hand, participants with an age range between 30 and 34 years (both included) were classified as <34 years (“YAG”—Younger Adults Group), and on the other hand, participants with an age range between 35 and 45 years (both included) were classified as persons > 34 years of age (“OAG”—Older Adults Group). The mean and standard deviation (SD) of the overall scores were then calculated for each of the OMES-E test variables. Subsequently, a comparison of means was made by means of the Student t-test for independent samples for each of the variables of that test, taking into account the two age groups. Then, for the qualitative variables of the clinical history and myofunctional examination test (MBGR), a cross-table was generated with the categorized age and each one of the blocks of variables that were classified for that test, using the chi-square test and Bonferroni-corrected z-test to establish which groups or subgroups were different from the rest. In turn, a comparison of means was made using the Student *t*-test for independent samples for the variables salivary pH, chewing time, oxygen saturation and heart rate before ingestion. Finally, a general linear model of repeated measurements was generated for the variables heart rate and oxygen saturation as a function of time and age. A significance level of *p* < 0.05 was established, applying a 95% confidence limit (95% CL).

## 3. Results

Table 1 shows the characteristics of the sample. Finally, there were 18 participants with DS (eight women and 10 men) with a mean age of 34.5. The mean body mass was 61.85 kg, and the mean height was 153.91 cm, so the mean body mass index (BMI) was 26.11. Of the total number of participants, four lived independently and 14 lived with their families.

Table 2 shows the results of health, respiratory and sleep problems in adults with Down syndrome categorized by age. In relation to health problems, there were no significant differences between the two groups, with *orthopedic problems* (62.5%) in the YAG and *digestive problems* (70%) in the OAG being the most frequent. Likewise, there were also no statistically significant differences in the respiratory problems category in both age groups. With respect to sleep problems, significant results were only obtained for *drooling during sleep* (*p* = 0.020), with a 10% difference in *drooling on a regular basis*, this frequency being higher in the YAG. Likewise, there were differences of 50% between both groups in *sporadic drooling*, with the highest figures corresponding to the OAG. Moreover, differences of 55% were observed between the age groups, being higher in the OAG in terms *of not* *drooling during sleep.*

Table 3 shows the results of feeding, chewing and swallowing problems, as well as oral and postural habit problems in adults with Down syndrome. Regarding feeding problems, no significant differences were obtained according to age. However, it is important to highlight the great difficulty in *introducing new* *flavors* *and consistencies* in both groups. Specifically, difficulties in the *introduction of new flavors* appeared in more than 60% of the participants and increased in more than 75% in the *introduction of new consistencies*. Regarding chewing problems, there were no statistically significant differences in the variables studied in both age groups, with the exception of the variable *deficient* *chewing capacity* (*p* = 0.034), where a difference of 37.5% was obtained in the age groups studied, being more pronounced in the YAG. Similarly, it is important to note that 100% of the participants reported *unilateral* *chewing*. Regarding swallowing problems, no statistically significant differences were obtained according to the age of the participants, but there was a high frequency (70–100% for OAG and YAG, respectively) of *food* *residues after swallowing* in both age groups. Within oral and postural habits, no statistically significant differences were found between age groups, although the YAG obtained higher percentages related to the presence of bad habits compared to the OAG, specifically a higher presence of *finger sucking, lip wetting, bruxism,* *dental pressure, nail biting* and *object biting.*

Table 4 shows the merged results of the supplementary tables regarding OMES-E total scores out of 100 for facial segments, facial mobility and swallowing problems in age-categorized adults with Down syndrome. Since the merged scores out of 100 did not yield significant differences, we have included those subsections within the corresponding category in which statistically significant differences were obtained. Along these lines, differences between groups were only found in the facial segments category in the mandible/maxilla variable (Supplementary Table S1), which showed significant results (*p* = 0.021) according to the age of the participants in the mandible/maxilla, specifically with regard to the anteroposterior relationship. The YAG obtained a mean score correlating with mild–moderate dysfunction, while the OAG showed moderate–severe dysfunction. Similarly, it should be noted that the palate category obtained the lowest (and therefore poorest) scores of all the variables, being associated with a *high* and *narrow palate*.

In reference to facial mobility (Supplementary Table S2), no significant results were obtained in any of the study variables according to the age of the participants, with the exception of the *upwards* and *downwards* lingual movements (*p* = 0.025). In relation to the overall scores for all facial mobility, the lowest scores out of 100 were obtained in reference to the cheek mobility variable.

Regarding swallowing problems and masticatory efficiency (Supplementary Table S3), no significant scores were obtained between age groups, with the lowest score out of 100 in the category of *swallowing efficiency*, with a lower mean for handling solids than liquids.

Table 5 shows the oximetry values, evidencing significant differences (*p* = 0.044) in the variable SaO_2_ during food intake, with the OAG presenting lower values. On the other hand, as for the heart rate values, no significant differences were obtained between age groups, obtaining mean heart rate scores between 73.6 for the YAG and 65.9–78.5 for the OAG.

## 4. Discussion

The present study aimed to analyze the state of the maxillomandibular and oral structure, masticatory and swallowing performance and eating and dietary habits in a group of adults with DS. The main results of the present study demonstrated the high frequency of digestive problems in adults with DS. Likewise, an important presence of problems related to the introduction of new flavors and consistencies was observed, limiting the foods included in their diet. In relation to chewing, unilateral chewing was reported, categorized as poor chewing. A condition of severe anatomical dysfunction was also observed in the mandibular–maxillary relationship, with poor oral muscular mobility influenced by a high presence of hypotonicity reflected in poor swallowing efficiency with solids. In spite of this, it was observed that, in general, these difficulties did not increase significantly beyond 34 years of age.

Previous studies have shown a number of medical comorbidities to be present in people with DS from birth, including a high prevalence of digestive disorders [25]. Specifically, 4.7% of all adults with DS present digestive problems, which are related to feeding difficulties caused by chewing and swallowing problems [10]. These parameters are positively correlated to the changes observed during physiological aging of the esophagus, manifesting as a decrease in esophageal sensation and alterations in upper esophageal sphincter contractility that can lead to the abovementioned digestive problems [26]. Along these lines, our study has also evidenced a high presence of digestive problems in the population with DS.

In relation to food, our study found the population with DS to have a high propensity to present difficulties both in the introduction of new flavors and in the introduction of new consistencies to their habitual diet. These results are in line with studies suggesting a direct relationship with the presence of reduced sensory awareness and the lack of teeth. This complicates chewing and causes persons with DS to be less inclined to expand the number of taste experiences in terms of flavors and consistencies, limiting them to those that are known and comfortable to handle within the oral cavity [27]. Along these lines, several studies express concern about feeding in the DS population, as the presence of oral and/or pharyngeal difficulties that jeopardize the safety and efficacy of swallowing can lead to life-threatening conditions such as malnutrition, dehydration and aspiration pneumonia [26]. Considering the aforementioned dental and masticatory problems, as well as their influence on swallowing, feeding and digestion, it is important to examine more closely the relationship between masticatory problems and dental malocclusions found in a large percentage of adults with DS [12]. Furthermore, these data correlate positively with the unilateral chewing pattern observed in the entire DS population studied [28].

In addition, people with DS have a characteristic cranial morphology, with typical alterations at the oral level. The mandibular canal can present anatomical variations that influence development of the jaw and the appearance of periodontal diseases, which in turn affect chewing function [29]. These observations coincide with the dysfunctions found in our study in the anteroposterior relationship between the mandible and maxilla. This causes an apparently larger tongue to protrude from a smaller mouth, giving rise to the now obsolete term of “macroglossia” in this particular population [30]. All of these oral disorders that influence masticatory function can also affect swallowing function. Specifically, it has been observed that most of the difficulties are associated with the oral phase of swallowing, with hypotonicity being one of the main factors, as it interferes with lingual and labial mobility [10]. These observations coincide with the results of our own study, in which a great difficulty in tongue mobility was recorded, especially in relation to the ascending and descending movements necessary for the correct formation of the bolus. Compensatory movements of other parts of the body were also recorded to facilitate the act of swallowing.

Finally, taking into account the risks posed by swallowing difficulties, oximetric parameters such as heart rate and blood oxygen saturation should be mentioned. Previous studies have evidenced the trend toward increasingly poorer cardiovascular capacity with aging [31]. These findings are in line with the results obtained in our study, where there is significant evidence of a reduction in SaO_2_ during swallowing in older participants. On the other hand, there are also studies that show significant changes in SaO_2_ in people with different disabilities, but in phases different from those found in our study, since they speak of an abrupt decrease after oral feeding in a seated position (and not during), which could be due to the posture itself, the presence of aspiration, fatigue in the swallowing process or apnea during swallowing. These same studies, in line with our results, also found no statistically significant differences in the cardiac pulse of the population with disabilities studied [32,33].

It is important to note that, except for the variables described above, we did not register significant differences with a cut-off value of 34 years, setting the minimum age limit at 30 and the maximum at 45. However, there are other studies, with different results, reporting an increase in the gradual decline of chewing and swallowing functionality as a function of age, as well as feeding, leading to an increase in associated complications [26]. Although our data are in contradiction with these previous investigations, it is important to highlight the small number of participants included for each established age range.

Regarding the limitations found in our study, the small sample size stands out, as well as the non-inclusion of a control group without DS, which makes it difficult to relate variables in a generalized and meaningful way to ensure a representative distribution for the DS population. Likewise, another limitation is the small number of previous investigations in the adult population with DS, since the vast majority of studies have been focused on the pediatric population.

## 5. Conclusions

After analysis of the results obtained in the selected assessment tests, it is concluded that there is a high frequency of digestive problems, a generalized refusal to introduce new tastes and consistencies, dysfunctional oral habits, poor chewing and swallowing patterns and oral hypotonia in the DS population. Importantly, this frequency of occurrence did not increase significantly when comparing the 30–34-year-old group (YAG) and the 35–45-year-old group (OAG). Therefore, it would be interesting to investigate whether these difficulties are maintained over time or increase in frequency of occurrence from another age range. In this regard, it is important to bear in mind that, from the age of 40–45 years, people with DS may present neuropathological changes related to Alzheimer’s disease (AD).

These results indicate the need to educate and raise awareness through specific intervention protocols for the younger generations of individuals with DS, as well as their family and social environment, underlining the importance of early interventions aimed at these oral dysfunctions, as they develop in childhood and are subsequently consolidated throughout life. These interventions would compensate for difficulties in oral functions to the extent that the anatomical characteristics of these individuals allow.

Further research is also needed in this field to identify other parameters that may affect chewing and swallowing function, as well as diet and nutritional status in adults with DS. These studies should include larger population samples, with older participants (>45 years) than those included in this study, in order to obtain meaningful results for the scientific community and contribute to improving the quality of life and autonomy of people with DS as aging progresses.

## Figures and Tables

**Table 1:** Sample characteristics.

Characteristics	Mean	Sd	Range
Age (years)	34.5	3.88	30–45
Body mass (kg)	61.8	8.79	42.8–79.8
Height (cm)	153.9	10.6	138–175
BMI (kg/m^2^)	26.1	3.03	21.5–31.2
Sex	**n**	**%**	
Female	8	40.0	
Male	10	60.0	
Age			
<34	11	61.1	
>34	7	38.8	
Independent life			
Yes	4	22.2	
No	14	77.8	

Sd: Standard deviation.

**Table 2:** Health, respiratory and sleep problems in adults with Down syndrome according to their environment.

Characteristics	YAG		OAG		*p*-Value
	n	%	n	%	
**Health problems**					
Neurological	2	25	0	0	0.183
Orthopedic	5	62.5	3	30	0.184
Metabolic	3	37.5	2	20	0.382
Digestive	2	25	7	70	0.077
Hormonal	2	25	1	10	0.412
**Respiratory problems**					
Frequent colds	2	25	4	40	0.437
Tonsillitis	1	12.5	1	10	0.706
Halitosis	0	0	1	10	0.556
Asthma	0	0	1	10	0.556
Rhinitis	1	12.5	0	0	0.444
Sinusitis	0	0	1	10	0.556
Nasal congestion	0	0	1	10	0.556
Nasal itching	4	50	7	70	0.352
Coryza	0	0	2	20	0.294
**Sleep problems**					
Restless sleep	3	37.5	1	10	0.206
Fragmented sleep	3	37.5	5	50	0.596
Noisy breathing during sleep	5	62.5	6	60	0.914
Snoring	6	75	5	50	0.502
Drooling during sleep	2	20	8	80	0.020
Apnea during sleep	1	12.5	2	20	0.588
Water intake at night	1	12.5	1	10	0.671
Open mouth during sleep	7	87.5	10	100	0.250
Dry mouth on waking up	7	87.5	10	100	0.556
Facial pain on waking up	0	0	1	10	0.357
Body position during sleep (lateral decubitus)	4	50	9	90	0.060
Body position during sleep (dorsal decubitus)	2	25	0	0	0.094
Body position during sleep (ventral decubitus)	2	25	1	10	0.396
Hand resting of face	2	25	2	20	0.800

YAG: younger adults group; OAG: older adults group.

**Table 3:** Feeding problems, chewing behavior, swallowing problems and oral habits in adults with Down syndrome.

Characteristics	YAG		OAG		*p*-Value
	n	%	n	%	
**Feeding problems**					
Difficulty introducing drinking glass	6	75	6	60	0.437
Difficulty introducing flavors	7	87.5	6	60	0.225
Difficulty introducing consistencies	6	75	8	80	0.618
Non-sweetened food	8	100	10	100	-
Sweetened foods	7	87.5	10	100	0.352
General form of food intake (pasty texture)	3	37.5	1	10	0.163
General form of food intake (solids)	5	62.5	9	90	0.163
**Chewing behavior**					
Chewing side (unilateral)	8	100	10	100	-
Lip during chewing (partly open)	5	62.5	4	40	0.343
Lip during chewing (open)	1	12.5	3	30	0.375
Noisy chewing	6	75	6	60	0.502
Liquid intake during chewing	8	100	8	80	0.180
Temporomandibular joint sounds	2	25	1	10	0.396
Chewing difficulty	3	37.5	3	30	0.737
Leaking from mouth during chewing	2	25	4	40	0.502
Chewing of food little	2	25	6	60	0.138
Chewing of food adequate	3	37.5	3	30	0.737
Chewing of food a lot	3	37.5	1	10	0.163
Chewing speed compared with family (slow)	4	50	4	40	0.671
Chewing speed compared with family (similar)	2	25	2	20	0.800
Chewing speed compared with family (fast)	2	25	4	40	0.502
Chewing capacity (good)	3	37.5	1	10	0.09
Chewing capacity (deficient)	3	37.5	0	0	0.034
Chewing capacity (regular)	5	62.5	7	70	0.737
**Swallowing problems**					
Swallowing difficulty	1	12.5	3	30	0.375
Noisy difficulty	2	25	4	40	0.610
Gagging during swallowing	1	12.5	2	20	0.652
Pain during swallowing	0	0	1	10	0.556
Reflux after swallowing	2	25	1	10	0.412
Leaking form mouth during swallowing	4	50	1	10	0.060
Throat clearing after swallowing	2	25	3	30	0.618
Cough after swallowing	4	50	6	60	0.520
Food residues after swallowing	8	100	7	70	0.090
**Oral habits**					
Sucking	3	37.5	3	30	0.737
Fingering	2	25	2	20	0.618
Tongue sucking	1	12.5	2	20	0.588
Wetting of lips	2	25	2	20	0.618
Bruxism	4	50	3	30	0.352
Dental pressure	6	75	3	30	0.077
Nail biting	2	25	1	10	0.412
Biting of oral mucosa	0	0	1	10	0.556
Biting of objects	2	25	0	0	0.183
**Postural habits**					
Lip biting	3	37.5	3	30	0.563
Mandibular protrusion	0	0	3	30	0.147
Hand resting of jaw	1	12.5	0	0	0.444
Hand resting of head	1	12.5	0	0	0.444

YAG: younger adults group; OAG: older adults group.

**Table 4:** OMES-E total scores out of 100 in adults with Down syndrome.

	YAG		OAG		*p*-Value
	Mean	Sd	Mean	Sd	
**Facial segments**					
Face	62.5	12.6	58.3	10.3	0.453
Cheeks	70.3	14.8	63.7	7.10	0.233
Mandible/maxilla	66.6	10.9	57.5	13.2	0.136
Anteroposterior relationship	2.88	1.13	1.80	0.63	0.021
Lips	66.6	10.9	57.5	13.2	0.136
Chin muscle	81.2	22.1	62.5	29.4	0.155
Tongue	65.6	12.9	66.3	15.6	0.929
Palate	53.1	17.3	50.0	19.5	0.728
**Facial mobility**					
Lip mobility	54.1	16.5	64.1	21.2	0.292
Tongue mobility	91.7	9.8	96.9	5.3	0.164
Upwards	4.5	1.8	64.1	21.2	0.292
Downwards	4.5	1.8	5.9	0.3	0.025
Check mobility	37.0	18.3	49.2	22.7	0.237
Mandibular mobility	69.5	15.1	82.3	19.5	0.149
Breathing	71.8	20.8	65.0	21.1	0.500
**Swallowing problems**					
Lip problems during swallowing	62.5	7.71	51.6	16.5	0.109
Tongue problems during swallowing	87.5	0.0	85.0	7.9	0.387
Problems during swallowing	79.1	6.29	80.0	9.78	0.838
Swallowing efficiency	47.2	7.86	46.6	7.03	0.876
Chewing bite	87.5	18.9	77.5	14.2	0.217
Chewing	52.5	10.3	50.0	10.5	0.621
Problems during chewing	72.9	17.6	76.6	19.5	0.679

YAG: younger adults group; OAG: older adults group; Sd: standard deviation. For OMES-E total scores, the subsections that obtained comparatively statistically significant differences between groups *p* < 0.05 were included. The rest of the evaluated items are shown in the Supplementary Materials.

**Table 5:** Salivary pH, chewing time, heart rate and SaO_2_ in adults with Down syndrome as a function of age.

Oximetry Values	YAG		OAG		*p*-Value
	Mean	Sd	Mean	Sd	
Salivary pH	6.19	0.70	6.15	0.88	0.308
Chewing time (minutes)	2.92	1.90	2.14	1.01	0.455
Heart rate (bpm)					
Before intake	73.6	13.30	65.9	14.2	0.529
During intake	76.3	9.33	78.5	16.3	0.349
After intake	78.5	16.3	63.7	10.9	0.455
SaO_2_ (mmHg)					
Before intake	96.2	1.09	94.0	3.89	0.372
During intake	96.0	2.49	95.7	2.06	0.044
After intake	96.1	1.35	96.3	2.26	0.261

YAG: younger adults group; OAG: older adults group; Sd: standard deviation.

## Data Availability

The data that support the findings of this study are available from the corresponding author J.M.-L., upon reasonable request.

## Supplementary Materials

The following are available online at https://www.mdpi.com/article/10.3390/ijerph19052616/s1, Table S1, OMES-E scores referred to facial segments in adults with Down syndrome. Table S2, OMES-E scores referred to facial mobility in adults with Down syndrome. Table S3, OMES-E scores referred to swallowing problems and chewing efficiency in Down syndrome. The following are available online at Cañizares-Prado, S.; Molina-López, J.; Moya, M. de la T.; Planells, E. Oral Function and Eating Habit Problems in People with Down Syndrome. 2021, doi:10.5281/zenodo.5732027.
