# Supplementary material for: Oral Function and Eating Habit Problems in People with Down Syndrome
Source: Int J Environ Res Public Health. 2022 Feb 24;19(5):2616. doi: 10.3390/ijerph19052616 (PMC8909609; doi:10.3390/ijerph19052616)
Supplement: Supplementary file 1 [file ijerph-19-02616-s001.zip › Suppl. Table S1. OMES-E scores referred to facial segments in adults with Down syndrome.pdf]

Suppl.Table S1. OMES-E scores referred to facial segments in adults with Down syndrome.

	YAG		OAG		*P-value*
	Mean	SD	Mean	SD	
**Face**					
Facial symmetry	2.88	0.84	2.70	0.68	0.629
Proportion two-thirds of the face	2.50	0.54	2.30	0.48	0.417
Nasolabial folds	2.13	0.35	2.00	0.47	0.543
Total score out of 100	*62.5*	*12.6*	*58.3*	*10.3*	0.453
**Cheeks**					
Cheek volume	3.13	0.84	3.10	0.57	0.941
Cheek tone / configuration	2.50	1.07	2.00	0.00	0.155
Total score out of 100	*70.3*	*14.8*	*63.7*	*7.10*	0.233
**Mandible / maxilla**					
Mouth open > 4 mm	2.75	0.46	2.50	0.71	0.402
Anteroposterior relationship	2.88	1.13	1.80	0.63	0.021
Midline relationship	2.38	0.52	2.60	0.70	0.460
Total score out of 100	*66.6*	*10.9*	*57.5*	*13.2*	0.136
**Lips**					
Resting lip function	3.13	0.84	2.70	0.95	0.335
Volume and configuration	2.25	0.46	2.50	0.53	0.307
Lip commissures	2.75	0.89	2.50	0.71	0.514
Lower lip rhyming	2.75	0.71	2.70	0.68	0.880
Total score out of 100	*66.6*	*10.9*	*57.5*	*13.2*	0.136
**Chin muscle**					
Chin muscle	3.25	0.89	2.50	1.18	0.155
Total score out of 100	*81.2*	*22.1*	*62.5*	*29.4*	0.155
**Tongue**					
Position and appearance	2.5	0.9	2.2	0.6	0.426
Appearance and volume	2.8	0.7	3.1	0.9	0.374
Total score out of 100	65.6	12.9	66.3	15.6	0.929
**Palate**					
Width	2.25	0.71	2.10	0.88	0.700
Height	2.00	0.76	1.90	0.74	0.781
Total score out of 100	*53.1*	*17.3*	*50.0*	*19.5*	0.728

YAG: Younger adults' group; OAG: Older adults' group; Sd: standard deviation.
