# Supplementary material for: Oral Function and Eating Habit Problems in People with Down Syndrome
Source: Int J Environ Res Public Health. 2022 Feb 24;19(5):2616. doi: 10.3390/ijerph19052616 (PMC8909609; doi:10.3390/ijerph19052616)
Supplement: Supplementary file 1 [file ijerph-19-02616-s001.zip › Suppl. Table S2. OMES-E scores referred to facial mobility in adults with Down syndrome.pdf]

Suppl. Table S2. OMES-E scores referred to facial mobility in adults with Down syndrome.

	YAG		OAG		*P-value*
	Mean	SD	Mean	SD	
**Lip mobility**					
Protrusion	5.63	0.74	5.60	1.27	0.961
Retrusion	3.88	1.96	5.40	1.58	0.086
Lateralization to right	1.75	1.39	2.20	1.81	0.571
Lateralization to left	1.75	1.39	2.20	1.81	0.571
Total score out of 100	*54.1*	*16.5*	*64.1*	*21.2*	0.292
**Tongue mobility**					
Protrusion	6.0	0.0	5.8	0.6	0.387
Retrusion	6.0	0.0	5.9	0.3	0.387
Lateralization to right	6.0	0.0	5.7	0.7	0.229
Lateralization to left	6.0	0.0	5.7	0.7	0.229
Upwards	4.5	1.8	5.9	0.3	0.025
Downwards	4.5	1.8	5.9	0.3	0.025
Total score out of 100	*91.7*	*9.8*	*96.9*	*5.3*	0.164
**Cheek mobility**					
Insufflation	1.5	1.1	2.0	2.1	0.551
sucking	3.0	2.3	3.6	2.3	0.589
Retraction	3.4	2.1	3.6	2.3	0.146
Move air from one cheek to the other	1.0	0.0	1.3	0.9	0.387
Total score out of 100	*37.0*	*18.3*	*49.2*	*22.7*	0.237
**Mandibular mobility**					
Open	6.00	.000[a]	6.00	.000[a]	-
Close	6.00	0.00	5.90	0.32	0.387
Lateralization to right	2.25	1.75	3.90	2.18	0.102
Lateralization to left	2.25	1.75	3.90	2.18	0.102
Protrusion	4.38	2.20	5.00	1.63	0.498
Total score out of 100	*69.5*	*15.16*	*82.3*	*19.5*	0.149
**Breathing**					
Breathing	2.88	0.84	2.60	0.84	0.500
Total score out of 100	*71.8*	*20.8*	*65.0*	*21.08*	0.500

YAG: Younger adults' group; OAG: Older adults' group; Sd: standard deviation.
