# Supplementary material for: Oral Function and Eating Habit Problems in People with Down Syndrome
Source: Int J Environ Res Public Health. 2022 Feb 24;19(5):2616. doi: 10.3390/ijerph19052616 (PMC8909609; doi:10.3390/ijerph19052616)
Supplement: Supplementary file 1 [file ijerph-19-02616-s001.zip › Suppl. Table S3. OMES-E scores referred to swallowing problems and chewing efficiency in Down syndrome.pdf]

Suppl. Table S3. OMES-E scores referred to swallowing problems and chewing efficiency in Down syndrome.

	YAG		OAG		*P-value*
	Mean	SD	Mean	SD	
**Lip problems during swallowing**					
Lip sealing	3.75	0.46	3.10	0.99	0.109
Total score out of 100	*62.5*	*7.71*	*51.6*	*16.5*	0.109
**Tongue problems during swallowing**					
Contained within oral cavity	4.0	0.0	3.9	0.3	0.387
Between the teeth	3.0	0.0	2.9	0.3	0.387
Total score out of 100	*87.5*	*0.0*	*85.0*	*7.9*	0.387
**Problems during swallowing**					
Associated movements	1.5	0.53	1.5	0.52	10.00
Sliding of mandible	1.38	0.51	1.2	0.42	0.440
Facial muscle tension	1.0	0.10	1.2	0.42	0.201
Food leakage	1.75	0.46	1.8	0.42	0.814
Cough	2.00	0.00	2.00	0.00	10.00
Noise	1.88	0.35	1.9	0.31	0.876
Total score out of 100	*79.1*	*6.29*	*80*	*9.78*	0.838
**Swallowing efficiency**					
Solids swallowing efficiency	1.63	0.52	1.30	0.48	0.188
Liquids swallowing efficiency	2.63	0.52	2.90	0.32	0.183
Total score out of 100	*47.2*	*7.86*	*46.6*	*7.03*	0.876
**Chewing - bite**					
First bite	3.5	0.8	3.1	0.6	0.217
Total score out of 100	*87.5*	*18.9*	*77.5*	*14.2*	0.217
**Chewing**					
Type of chewing	5.25	1.04	5.00	1.05	0.621
Total score out of 100	*52.5*	*10.3*	*50.0*	*10.5*	0.621
**Problems during chewing**					
Associated movements	1.38	0.52	1.40	0.52	0.920
Postural changes	1.63	0.52	1.70	0.48	0.755
Food leakage	1.38	0.52	1.50	0.71	0.682
Total score out of 100	*72.9*	*17.6*	*76.6*	*19.5*	0.679

YAG: Younger adults' group; OAG: Older adults' group; Sd: standard deviation.
